# Extended and Generic Higher-Order Elements for MEMS Modeling

**DOI:** 10.3390/s22208007

**Published:** 2022-10-20

**Authors:** Zdeněk Biolek, Viera Biolková, Dalibor Biolek, Zdeněk Kolka

**Affiliations:** 1 Department of Electrical Engineering, University of Defence, 662 10 Brno, Czech Republic; 2 Department of Microelectronics, Brno University of Technology, 616 00 Brno, Czech Republic; 3 Department of Radio Electronics, Brno University of Technology, 616 00 Brno, Czech Republic

**Keywords:** Chua’s table, higher-order element, extended element, generic element, pinched hysteresis loop

## Abstract

State-dependent resistors, capacitors, and inductors are a common part of many smart engineering solutions, e.g., in MEMS (Micro-Electro-Mechanical Systems) sensors and actuators, Micro/NanoMachines, or biomimetic systems. These memory elements are today modeled as generic and extended memristors (MR), memcapacitors (MC), and meminductors (ML), which are more general versions of classical MR, MC, and ML from the infinite set of the fundamental elements of electrical engineering, known as Higher-Order Elements (HOEs). It turns out that models of many complex phenomena in MEMS cannot be constructed only from classical and state-dependent elements such as R, L, and C, but that other HOEs with generalized behavior should also be used. Thus, in this paper, generic and extended versions of HOEs are introduced, overcoming the existing limitation to MR, MC, and ML elements. The relevant circuit theorems are formulated, which generalize the well-known theorems of classical memory elements, and their application to model complex processes of various physical natures in MEMS is shown.

## 1. Introduction

The increased demand for smart technical solutions makes great demands on the research and development of complex systems based on cooperation between the subsystems of different physical natures. Examples are MEMS, Micro/Nano Machines, or systems with biomimetic elements, which often ingeniously use various physical principles from the fields of electrical engineering, electronics, mechanics, optics, and thermics, but also microfluidic systems, biology, etc., in a single application. Resistor, capacitor, and inductor elements are commonly used as building blocks for modeling non-linear phenomena of various physical natures. These three elements represent the basic constitutive relations between quantities of current, electrostatic, and magnetic fields.

Circuit theory and modern electronics are completely dependent on the concept of resistors, capacitors, and inductors, despite the fact that these ideal elements do not occur in the real world. Their use in describing reality is a necessity since the constitutive relations of these elements are expressions of the fundamental laws governing reality. This trinity was extended in 1971 by the memristor [1] and in 1980 by other members of the family of general (*α*,*β*) elements [2], also known as Higher-Order Elements, HOEs. The HOEs are one-ports characterized by a unique constitutive relation between the quantities *v*^(*α*)^ and *i*^(*β*)^, where *v* and *i* are the voltages at the terminals of the element and the current flowing through the element. Positive indices denote the order of differentiation and negative indices the order of integration with respect to time. Resistors, capacitors, inductors, and memristors thus become special cases of (*α*,*β*) elements denoted by (0,0), (0,−1), (−1,0), and (−1,−1). The representation of elements as points in the *α* − *β* plane was taken as Chua’s periodic table of the basic elements of electrical engineering [2].

The resistor, capacitor, inductor, and other HOEs are just some of the pieces of the puzzle we use to try to create as accurate a model as possible of what actually happens in real systems. Of course, this approach has its limitations, which have already been described in the original paper [2]. The so-called memristive [3], memcapacitive, and meminductive systems [4] are much closer to the real manifestations of resistance, capacitance, and inductance. These are state-dependent resistors, capacitors, and inductors, aka generic memristors, memcapacitors, and meminductors. In addition, if these one-ports exhibit nonlinearity with respect to their constitutive quantities (voltage/current, charge/voltage, current/flux), they are referred to as extended memristors, memcapacitors, and meminductors [5].

With the introduction of (*α*,*β*) elements, the era of so-called predictive modeling begins. In general, the nonlinear constitutive relation of the element

(1) $$F\left(v^{\left(\alpha \right)},i^{\left(\beta \right)}\right)=0$$

is maintained unconditionally regardless of the circumstances in the network which the element is connected to. Each element type is fundamental in the sense that HOEs with a particular combination of the indices (*α*,*β*) cannot be replaced by any combination of HOEs with other indices (α*,β*). By linking HOEs in a variety of topologies, a diverse range of manifestations of nonlinear dynamics can be mimicked. Due to the principle of the universal validity of the nonlinear constitutive relation, the rule “one model for all situations” applies. Based on physical analogies, Chua’s table has also been generalized for elements of a non-electrical nature [6], opening the way for predictive modeling in interdisciplinary fields. For example, its application to the modeling of MEMS, which use various interconnected physical principles, is interesting. Silicon nanowires, which exhibit memristive properties, have started to be used as sensors of various physical quantities. These include gas sensors [7], silicon nanowire devices functionalized with rabbit antibodies in order to sense antigens [8], ion-sensitive FETs for cancer makers [9], or for DNA detection [10]. The work [11] introduces the concept of memsensors, a class of electronic devices in which the combination of sensing a physical quantity and memory resembles the dynamic response of biological systems to the environment and to stimuli. The UV memsensor is introduced in [12]. The interesting papers [13,14] are devoted to memristive and memcapacitive processes occurring in biomimetic membranes. The work [15] analyzes the memristive properties of microtubules as important components of the promising concept of submolecular computers. Paper [16] reports on a microfluidics-based design for four fundamental circuit elements in electronics, namely the resistor, inductor, capacitor, and memristor.

Extended and generic memelements can be characterized by a unified notation of the port and state equation

(2a) $$y=P\left(x,u\right)u,\;\dot{x}=f\left(x,u\right),$$

where *y* and *u* are the constitutive variables, ***x*** is generally a state vector, and *P*() is a one-port parameter satisfying the condition

(2b) $$\underset{u\to 0}{\lim}P\left(x,u\right)\neq \infty$$

for each state ***x***. Generic memelements have a parameter that depends only on the state ***x***, i.e., *P*() = *P*(***x***). The meaning of the quantities *y* and *u* associated with each element is illustrated in Table 1. A schematic symbol is added to each element type. The indices *e* and *g* denote the extended and generic element, respectively, and distinguish this element from the ideal element.

The basic fingerprint of any (*α*,*β*) element is a unique trace of the constitutive relation (1), along which the operating point moves under any circumferential situation in the plane of constitutive quantities *v*^(*α*)^ − *i*^(*β*)^. However, the best-known fingerprint is the so-called Pinched Hysteresis Loop (PHL), which arises in the *v*^(*α*+1)^ − *i*^(*β*+1)^ plane during bipolar excitation of the element [17]. The *v* − *i* PHLs appearing during the harmonic excitation of a memristor are very well known. Less known is, for example, the fact that PHLs are also plotted in the *v*^(1)^ − *i*^(1)^ plane during bipolar excitation of an arbitrary nonlinear resistor [17].

Note that, for example, the *ideal* memristor as an element (−1,−1), preserving the constitutive relation between the flux*φ* = *v*^(^^−1)^ and the charge *q* = *i*^(−1)^, i.e., being defined in the *v*^(−1)^ − *i*^(−1)^ plane, the *extended* and *generic* memristors are defined in the *v*^(0)^ − *i*^(0)^ plane by the relation (2) between voltage and current. The last row of Table 1 is a generalization of this reasoning to *extended* and *generic* (*α*,*β*) elements. These elements do not provide unambiguous constitutive relations as the ideal (*α*,*β*) elements do, but they are defined by the relations between the quantities *v*^(*α*+1)^ and *i*^(*β*+1)^, i.e., in a plane in which, the same as for ideal HOEs, PHLs occur. The existence of this kind of hysteresis in the *y–u* plane is guaranteed by (2) for finite values of the parameter *P*().

Fingerprints such as the PHLs and some others help to correctly identify an element or process and are a practical aid in evaluating model reliability. This is the basis for the methodology of predictive modeling used in [18] for memcapacitive MEMS and memcapacitive biomimetic systems or in [19] for electromechanical meminductive systems.

The aim of this paper is to present a new method for modeling processes in which the fingerprints of generic or extended elements, which are neither memristors, memcapacitors, or meminductors, can be identified. The theoretical basis necessary to recognize specific types of extended and generic HOEs by the fingerprints they leave in existing processes, regardless of their physical nature, is presented. The model can then be synthesized exclusively from these HOEs, possibly using their multi-port variants [18,19].

The fingerprints of extended and generic HOEs are discussed in Section 2 of this work. It is a generalization of some results that have so far been published for the cases of memristors or memcapacitors and meminductors. Some of the results are new and have not been published before. In Section 3, an example from the field of fluid mechanics is presented, including a predictive SPICE model together with simulation results. This application shows how the description of interdependent processes can be approached by methods of predictive modeling.

## 2. Fingerprints of Extended and Generic HOEs

A number of fingerprints of the extended memristors, previously known as the memristive systems [3], are well described in the literature. Some of these fingerprints have been generalized to extended memcapacitors and meminductors [4]. A glance at Table 1 and at the definition (2) shows the reason why such an extension is a simple matter: an extended and a generic memcapacitor or meminductor can be investigated in the same way as an extended or a generic memristor if we use charge and voltage (*q*,*v*) or flux and current (*φ*,*i*) instead of the voltage and current variables (*v*,*i*). The formally identical equations of motion allow us to generalize via physical analogy most of the known fingerprints of extended and generic memristors to generic extended and generic HOEs as well if we use the generalized voltage *v*^(*α*)^ instead of voltage *v* and the generalized current *i*^(*β*)^ instead of current *i*.

### 2.1. Zero-Crossing Property

**Theorem 1.**  *Let it hold for each state **x**.*

(3) $$P\left(x,u\right)u=H\left(x,u\right),\;\underset{u\to 0}{\lim}H\left(x,u\right)=0.$$

*For any state **x,** let H(**x**,u) be a continuous function with respect to the variable u at point (**x**,0). Then, the sets of zero crossing times {t_k_(u)} and {t_k_(y)} of the excitation u and the corresponding response y are identical.*

**Proof.**  The boundary condition of assumption (3) implies that for every *u* = 0, *y* = 0 must also hold. The continuity of the function *H*() at *u* = 0 implies in turn that *u* = 0 for every *y* = 0. □

The zero-crossing property means that for extended and generic (*α*,*β*) elements satisfying condition (3), the quantities *v*^(*α*+1)^ and *i*^(*β*+1)^ always cross the zero level at the same time. Condition (3) differs from condition (2a) stated in the existing definition of extended elements, which allows only bounded values of the parameter *P*() at zero excitation. According to (3), an infinite value of the parameter is allowed if the product *P*()*u* is zero for *u* = 0. The case of an extended memristor with an infinite value of conductivity at zero excitation, which still exhibits the Zero crossing property, will be discussed in Section 3.

For extended and generic (−1,−1) memristors, the quantities voltage *v* and current *i* pass simultaneously through the zero crossing level. It is shown in [3] that the zero crossing property is guaranteed for passive memristors. This property also implies that a passive memristor cannot store energy. On the contrary, energy is stored by extended and generic (−1,−2) memcapacitors and (−2,−1) meminductors since the zero crossing property for these elements does not refer to voltage and current but to the pair of quantities (*v*,*i*^(−1)^) = (*v*,*q*) and (*v*^(−1)^*,i*) = (*φ,i*).

### 2.2. Pinched Hysteresis Loop

**Theorem 2.**  *Any excitation of an extended and generic (α,β) element according to (2) and (3) in which the excitation quantity v^(α+1)^ or i^(β+1)^ changes the sign results in hysteresis loops pinched at the v^(α+1)^ − i^(β+1)^ origin.*

**Proof.**  The hysteresis in the space of the excitation *u* and the response *y* results from the fact that, according to (2), the instantaneous value of *y* depends not only on the instantaneous value of *u* but also on the instantaneous value of the state ***x***. Thus, if the excitation *u* repeatedly passes through the same level, the quantity *y* generally has different values. The pinching of the hysteresis curves around the origin is a direct consequence of the Zero crossing property *2*.*1*. □

The PHL is often presented as a response to harmonic excitation. The loop gradually settles into a finite pattern as the quantity ***x***(*t*) converges to a periodical steady state. This is different from the ideal elements with the algebraic constitutive relation (1): there, a unique loop is formed instantaneously, i.e., already during the first period [20]. The paper [21] gives many examples of PHLs of various devices, some of which date back two centuries (discharge lamps, tungsten filaments, sodium or mercury tubes, electric arcs, thermistors). These are mostly cases for *α* = *β* = −1, today classified as extended and generic memristors. Loops can also be commonly observed in MEMS applications based on memcapacitive and meminductive principles [20]; they are also an inherent fingerprint in biomimetic [13,14] and microfluidic [16] systems.

The way that PHL is pinched at the origin divides PHLs into types I and II [22] or crossing type (CT) and non-crossing type (NCT) according to whether or not the present loop orientation changes after passing through the origin. In the case of tangential passage through the origin, where the two arms of the loop lobe touch each other, the order of touching *k* is observed. The loop is then of type CT(*k*) or NCT(*k*) [23,24].

**Figure 1:**
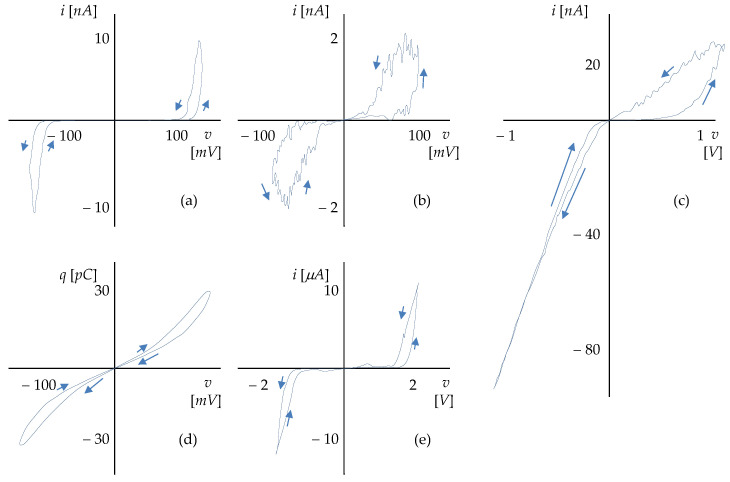
Different types of PHLs. (**a**,**b**) biomimetic memristors DPhPC and BTLE [13], (**c**) memristive UV sensor [11], (**d**) biomimetic membrane as a two-state memcapacitor [14], and (**e**) microtubular memristor [15].

Figure 1 shows examples of pinched PHLs of existing elements across different physical platforms. Cases (a) and (b) are the PHLs of biomimetic memristors based on DPhPC (diphytanoylphosphatidylcholine) and BTLE (brain total lipid extracts) [13] (NCT). Figure 1c shows the loop of a ZnO memristor [11] in a sensor of UV radiation (CT). Figure 1d shows the loop between charge and voltage on a biomimetic memcapacitance membrane formed between two lipid droplets [14] (NCT). The case in Figure 1e shows a PHL microtubule that is part of the cytoskeleton. It behaves, among other things, as a memristor [15] (NCT).

The type of loop is closely related to the physics of the processes that are responsible for the hysteresis. A brief analysis will be given in Section 2.4.

### 2.3. High-Frequency Behavior

**Theorem 3.**  *Let the system forming the dynamics of element (2) be bounded-input bounded-state (BIBS) stable. Furthermore, let the function **f**() from the definitional relation (2) be continuous with respect to the variables **x** and u. Then, the extended HOE (2) behaves under periodic high-frequency excitation as an ideal HOE with the constitutive relation.*

(4) $$y=P\left(x_{0},u\right)u$$

*where **x**_0_ is the limit state that the system tends to as the frequency increases.*

**Proof.**  BIBS stable systems [3] respond to bounded excitation *u* by a bounded state ***x***. Assuming the continuity of the function ***f***(), the rates of periodical changes of state will also be bounded. Thus, the amplitudes of the individual harmonic components of the state vector obtained by integrating the function ***f***() with respect to time must be inversely proportional to the frequency. With increasing excitation frequency, the system state inevitably tends to a constant state ***x***_0_. Thus, the change in the parameter *P*() at high frequencies is only due to the change in excitation *u*, as shown in relation (4). □

A detailed proof carried out for extended memristors can be found in [3]. As the frequency is increased, the hysteresis *y-u* curve gradually degenerates into a unique curve (4). The extended or generic memristor/memcapacitor/meminductor/(*α*,*β*) element becomes a nonlinear or linear resistor/capacitor/inductor/(*α* + 1,*β* + 1) element.

### 2.4. Type of PHL

Consider the PHL of element (2) in a steady state, excited by the signal

(5) $$u\left(t\right)=U\sin\left(\omega t\right),$$

where *U* is the amplitude and *ω* is the angular frequency. Suppose that the two arms of the PHL can be described in a certain neighborhood of the origin by the single-valued functions *y*^+^(*u*) and *y*^–^(*u*), where the indices + and—denote the loop arm along which the operating point travels from the first to the third and from the third to the first quadrant, respectively. Let us introduce the function [23]

(6) $$\Delta \left(u\right)=y^{+}\left(u\right)-y^{-}\left(u\right),$$

where the superscripts + and − denote the positive and negative arms of the loop, respectively. Let the function (6) have finite derivatives of all orders in the given neighborhood of the origin. Then, these derivatives at the point *u* = 0 contain information about the type of loop pinching. A crossing (CT) or non-crossing (NCT) type is decided by whether or not the function (6) changes the sign during this crossing.

Let us denote the difference between the *kth* order steepnesses of the two PHL arms at the *y–u* origin as

(7) $$\Delta_{k}=\underset{u\to 0}{\lim}\frac{d^{k}\Delta \left(u\right)}{du^{k}},$$

where *k* is a positive integer. Let *k_min_* be the smallest possible *k* for which ∆*_k_*≠ 0. Then, the following theorems hold:

**Theorem 4.**  *If k_min_ is an odd number, then the PHL is of class CT(k_min_ − 1).*

**Proof.**  If the order *k_min_* of the first nonzero derivative of the function (6) at the origin is an odd number, then (6) changes the sign at that point. This means that there is a crossing of the arms with a touch of order (*k_min_* − 1) when passing through the origin. □

**Theorem 5.**  *If k_min_ is an even number, then the PHL is of class NCT(k_min_ − 1).*

**Proof.**  If the order *k_min_* of the first nonzero derivative of the function (6) at the origin is an even number, then (6) does not change the sign at that point. This means that there is no crossing of the arms when passing through the origin and the order of their touching is (*k_min_* − 1). □

Let us suggest a way to obtain a particular form of (7) for *k* = 1, 2, and 3. Considering (2), the derivatives in (7) can be rewritten in the form

(8) $$\Delta_{k}=\underset{u\to 0}{\lim}{\left[k\frac{d^{k-1}P\left(x,u\right)}{du^{k-1}}+\frac{d^{k}P\left(x,u\right)}{du^{k}}u\right]}_{-}^{+},$$

where the indices going with the right square bracket mean that the content of the brackets for the negative arm of the loop is subtracted from the content of the brackets for the positive arm of the loop.

For *k* = 1, (8) will be of the form

(9) $$\Delta_{1}=\underset{u\to 0}{\lim}\left[P^{+}(x,u)-P^{-}(x,u)\right],$$

where the indices + and—denote the values of the *P*() parameter for the positive and negative arms of the loop. For *k* = 2, the derivative in the first term inside the square bracket (8) will be equal to

(10) $$\frac{dP}{du}=\frac{\partial P}{\partial u}+\frac{\partial P}{\partial x^{T}}\frac{dx}{du}=\frac{\partial P}{\partial u}+\frac{\partial P}{\partial x^{T}}\frac{\dot{x}}{\dot{u}}.$$

After a slight modification, ∆_2_ can be written as

(11) $$\Delta_{2}=\underset{u\to 0}{2\lim}\left[\frac{\partial P^{+}}{\partial u}-\frac{\partial P^{-}}{\partial u}+\frac{1}{U\omega }\left(\frac{\partial P^{+}}{\partial x^{T}}f^{+}+\frac{\partial P^{-}}{\partial x^{T}}f^{-}\right)\right],$$

where ***f*** = ***f***(***x***,*u*) is the nonlinear function from the definition (2), and the indices + and—denote the value of the function ***f***() for the positive and negative arms of the loop.

By analyzing (9) and(11), the following findings can be obtained.

**Theorem 6.**  *Let the lowest frequency of the harmonic component in the spectrum of steady-state response **x**(t) to the excitation (5) be an odd multiple of the excitation frequency ω. Then, the PHL is of type CT(1) except the case when*

(12) $$\underset{u\to 0}{\lim}P(x,u)=const$$

**Proof.**  In the case of an *odd* multiple of the excitation frequency, the steady state ***x***(*t*) has a different value at the beginning of each half-period of the excitation signal *u*(*t*) than at the end of the half-period. If the exception (12) does not apply, then the values of the parameter *P*(***x***,*u*) will also be different. This results in ∆_1_ ≠ 0, so that the two arms of the PHL form a non-zero angle at the origin of the coordinates, which indicates a crossover. □

If exception (12) applies, the loop type must be determined by further analysis. A similar analysis of (11) with respect to the validity of (12) then shows that the ∆_2_ term has a non-zero value, which would imply a loop of the NCT(1) type. The exception is the case where

(13) $$\underset{u\to 0}{\lim}\frac{\partial P}{\partial u}=const.$$

Further analysis would show that ∆_3_ = 0, which eliminates the possibility of a CT2-type crossing. This would be continued by testing the possibility of NCT(3) using ∆_4_.

Similarly, the next theorem applies:

**Theorem 7.**  *Let the lowest frequency of the harmonic component in the spectrum of steady-state response **x**(t) to the excitation (5) be an even multiple of the excitation frequency ω. If the following condition applies*

(14) $$\underset{u\to 0}{\lim}\frac{\partial P}{\partial x^{T}}f\neq 0,$$

*then the PHL will be of the NCT(1) type.*

**Proof.**  If the steady state ***x***(*t*) oscillates at an even multiple of the excitation frequency *u*(*t*), then its values will always be the same at the beginning and at the end of the half-period of the excitation signal. Therefore, the values of the parameter *P*() must also be the same, so ∆_1_ = 0. In addition, if the condition (14) holds, then ∆_2_ ≠ 0, which means that the PHL is a non-crossing type with the order of touching 1. □

Theorems 4 to 7 hold under the initial assumption that the functions *y*^+^ *(u*) and *y*^–^ *(u*) describing the two arms of the PHL have finite derivatives of arbitrary order around the origin. However, this condition is not always satisfied, as the following example shows. In such cases, one has to proceed judiciously. If there are appropriate limits on the left and right, one can work with the proposed methodology by finding the value of ∆*_k_* (7) separately for the first and third quadrants.

## 3. Illustrative Example—Fluid Meminerter

### 3.1. Device Analysis

**Figure 2:**
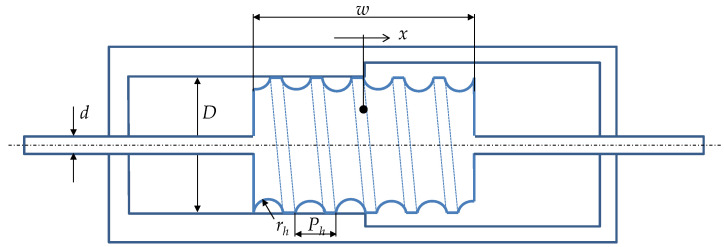
Fluid meminerter according to [27].

The inerter as a new mechanical element was proposed by Smith in [25]. The element greatly advances the possibilities of the synthesis of passive mechanical networks and has found its application as a shock absorber in the automotive industry, in the construction of earthquake-resistant buildings, etc. A fluidic version of the inerter is proposed in [26]. This device has been modified into a position-dependent inerter or meminerter in [27]. Its arrangement is presented in Figure 2.

In a cylinder of inner diameter *D* filled with a liquid of density*ρ*, a piston of width *w* moves, on the surface of which a helical channel with pitch *P_h_* is created for the conduction of the liquid. A channel with a semicircular cross-section of radius *r_h_* is formed only where the piston moves tightly in the cylinder, i.e., in the left half of its length; the channel is not formed in the second chamber because of the increased diameter of the cylinder. It is the active part of the helical channel that transfers the fluid between the left and right chambers by the movement of the piston. The movement of the fluid mass produces an inertial effect characterized by the inertial force *F_b_* or the momentum *p_b_* = ∫*F_b_* dt. The relationship between the momentum and the piston velocity is

(15) $$p_{b}\left(x,\dot{x}\right)=\underset{B\left(x\right)}{\underset{︸}{b_{0}\left(\frac{w}{2}-x\right)}}\dot{x},$$

where *B* is the inertance depending on the piston position *x*,

(16) $$b_{0}=\frac{\pi \rho {\left(D^{2}-d^{2}\right)}^{2}\sqrt{P_{h}^{2}+{\left(\pi D\right)}^{2}}}{8P_{h}r_{h}^{2}}.$$

According to [27], the piston should only move within the range of

(17) $$x\in \left(-\frac{w}{2},\frac{w}{2}\right).$$

Integrating both sides of equation (15) with respect to time yields an unambiguous relationship between the time integral of the momentum (15) and the piston position. Converting the integration with respect to time to an integration with respect to position *x*, and considering the bounds (17), the unambiguous constitutive relation will be

(18) $$\delta_{b}\left(x\right)=\int_{x_{0}}^{x}B\left(x^{\prime }\right)dx^{\prime },$$

where *x*_0_ is the initial piston position. For *x*_0_ = 0 and with respect to (15), the constitutive relation (18) has the form

(19) $$\delta_{b}\left(x\right)=\frac{b_{0}}{2}x\left(w-x\right).$$

The element with the constitutive relation (19) is denoted as a meminerter in [27]. For the electromechanical *F-i* or *F-v* analogy, it is a (−1, −2) memcapacitor or a (−2, −1) meminductor.

In the arrangement of Figure 2, parasitic damping is caused by viscous effects in the fluid. Losses occur in the flow through the helical channel, in the transitions between the two ends of the channel, and also in the viscous shear friction between the piston and the cylinder. After introducing simplifications in [27], the total damping force is

(20) $$F_{d}\left(x,\dot{x}\right)=\underset{C\left(x,\dot{x}\right)}{\underset{︸}{\left[\left(c_{1}\left(\frac{w}{2}-x\right)+c_{2}\right)\left|\dot{x}\right|\right]}}\dot{x},$$

where

(21a) $$c_{1}\approx 0.004283\frac{\pi \rho {\left(D^{2}-d^{2}\right)}^{3}\sqrt{P_{h}^{2}+{\left(\pi D\right)}^{2}}}{P_{h}r_{h}^{4}\sqrt{D_{h}R_{h}}},$$

(21b) $$c_{2}=\frac{3\pi \rho {\left(D^{2}-d^{2}\right)}^{3}}{64r_{h}^{4}},$$

*R_h_* is the bend radius of the helical channel, and *D_h_* is the hydraulic diameter of the channel [4]. Compared to [27], the absolute value for velocity is added to equation (20) to guarantee that the direction of the damping force depends on the direction of velocity. 

The element defined by (20) is referred to as an extended mem-dashpot in [27]. For the electromechanical *F-i* or *F-v* analogy, it is an extended memristor or an extended (−1,−1) element.

The friction at the interface of piston rods and seals is also taken into account in [27]. It has been shown experimentally that this is the Coulomb friction, which can be described by the relation

(22) $$F_{c}\left(\dot{x}\right)=f_{0}\mathrm{sgn}\left(\dot{x}\right),$$

where *f*_0_ is the magnitude of the frictional force, which is independent of the instantaneous velocity but always acts against the direction of motion.

For the electromechanical *F-i* or *F-v* analogy [28], the element (22) is a resistive element that can be modeled as a controlled current or voltage source.

### 3.2. Modeling and Simulation

**Figure 3:**
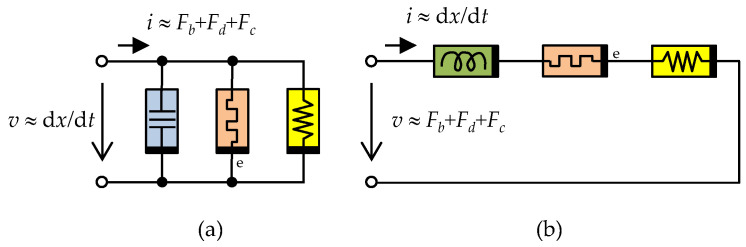
Electrical circuit diagrams of the devices in Figure 2 according to the electromechanical (**a**) *F*-*i*, (**b**) *F*-*v* analogy. Elements used: ideal memcapacitor (blue), ideal meminductor (green), extended memristor (red), and resistor (yellow).

The resulting force *F* acting between the cylinder and the moving piston in Figure 2 is the sum of the forces representing the inertia (19), viscous damping effects (20), and Coulomb friction (22). The equivalent electrical schematics of the entire device after applying the electromechanical *F-i* and *F-v* analogies according to Table 2 are presented in Figure 3.

The inertia effects are governed by the constitutive relation (19) between the mechanical quantities TIM and displacement, which corresponds, according to Table 2 and F-i /F-v analogy, to the constitutive relation between TIC and flux/TIF and the charge of an ideal memcapacitor/meminductor. Similarly, it can be concluded that viscous losses (20) will be modeled by an extended memristor, and Coulomb friction (22) by a resistor.

**Figure 4:**
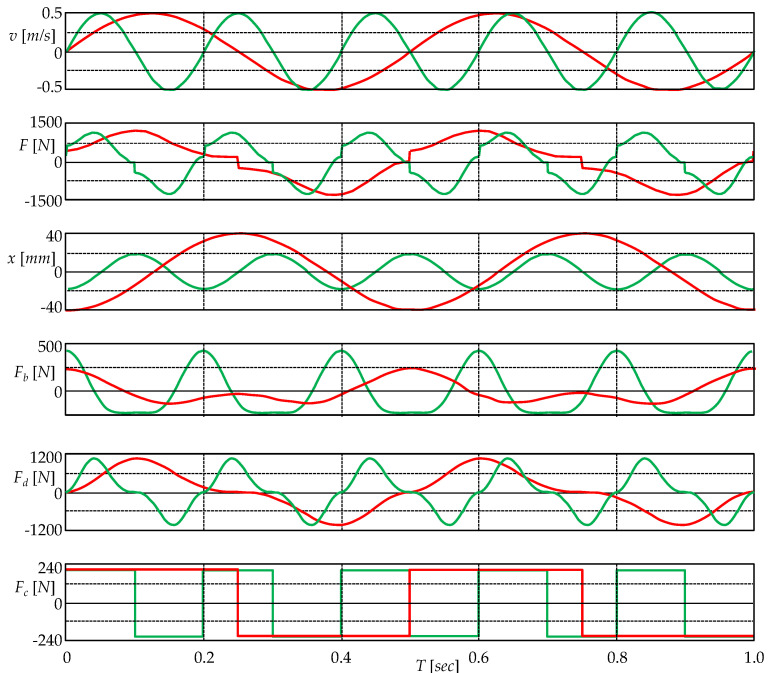
Waveforms of quantities during sinusoidal excitation of the device from Figure 2. From top to bottom: piston velocity *v* relative to the cylinder, total force *F* between the piston and cylinder, instantaneous position *x* of the piston center relative to the cylinder center, inertial force *F_b_* of the fluid as a derivative of the momentum (15) with respect to time, viscous friction force *F_d_* (20) in the fluid, Coulomb friction force *F_c_* (22).

The computer simulation is based on the arrangement in Figure 3b. The series connection of the three elements is driven by a sinusoidal current source that corresponds to the relative velocity of the piston with respect to the cylinder, with a magnitude of 0.5 m/s. The current excitation is designed to result in a harmonic motion of the piston around the cylinder center, see Figure 2. The device parameters are given in the SPICE model of the extended damper; see the Appendix A. The waveforms of piston velocity, the resultant force, the instantaneous piston position, and all three types of force acting in the device for excitation frequencies of 2 Hz and 5 Hz are plotted in Figure 4.

**Figure 5:**
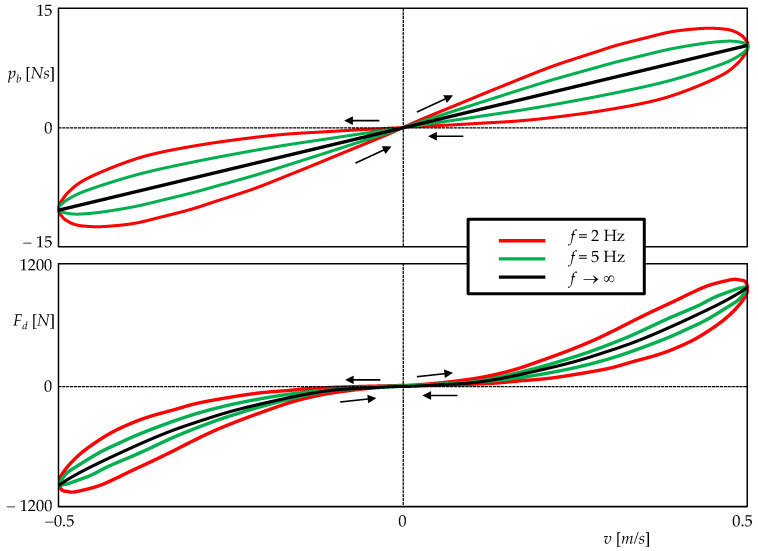
Hysteresis loops accompanying the phenomena of inertance (**top**) and viscous friction (**bottom**). Both loops are of type I, i.e., with a crossing at the origin. As the frequency increases, the hysteresis gradually disappears, and the loops turn into unambiguous curves.

Figure 5 shows the hysteresis loops characterizing the inertance and viscous damping processes for excitation frequencies of 2 Hz and 5 Hz. The inertance is truly modeled by an ideal meminductor, so the PHL must be of type I with the order of touching 0, i.e., CT(0) [29]. In addition, this type of crossing can be confirmed by the criteria given in Section 2.4. The function (15) is differentiable to all orders in the origin, so these criteria apply to it. The state *x* oscillates at the same frequency as the velocity (an odd multiple of the frequency) and (12) does not apply, indicating a type-I PHL with an apparent crossover, where both arms of the PHLs take a non-zero angle at the origin.

The simulation results at the bottom of Figure 5 show that viscous friction also leads to a PHL of type I. However, function (20) has a discontinuous second-order derivative with respect to the velocity at the origin, so the procedure of Section 2.4 cannot be applied routinely. A careful analysis of (9) and (11), distinguishing the limits on the left and right, leads to the conclusion that the PHL is of the NCT(1) type, i.e., the non-crossing type with the order of touching 1.

With increasing frequency, the loops gradually degenerate into unambiguous curves. The PHL of the inertia process becomes a line segment, while the PHL of the viscous friction process becomes a unique curve

(23) $$F_{d}=\left(c_{1}\frac{w}{2}+c_{2}\right)\mathrm{sgn}\left(\dot{x}\right){\dot{x}}^{2},\;\dot{x}=v,$$

which is consistent with the property 2.3. It is a quadratic parabola, one arm of which is rotated from the second to the third quadrant by changing the sign.

## 4. Discussion

This work provides a set of extended and generic (*α*,*β*) elements useful for modeling the processes of various physical natures. As part of the definition, we have changed condition (2a), which is still considered for extended memristors, memcapacitors, and meminductors, to the less restrictive condition (3). This is because, according to the current definition, an extended memristor cannot be, for example, a state-dependent resistor used to model the damping effects given by (20), even though this element reliably exhibits all the fingerprints of an extended memristor. Indeed, its memristance (memory resistance) is, according to (20), zero for zero current passing through, so it must be of infinite memductance (memory conductance) for zero terminal voltage (see the top section of Table 1) and thus violates the condition (2a). This condition is not satisfied by other provably memristive processes, e.g., fluid mechanics processes governed by the Drag equation [30] or memristive phenomena subject to the Mott–Gurney square law [31] for charge-carrier mobility in semiconducting thin-films. Compared to the existing (2a), the proposed condition (3) allows for an infinite value of the parameter *P*() while guaranteeing the zero-crossing property and other fingerprints of the extended element.

The theorems presented in this paper are generalized and modified versions of the theorems published so far for the cases of extended or generic memristors, memcapacitors, and meminductors. The basis for the proof of Theorem 1 (Zero crossing property) has been taken from [5], and the proof is modified with respect to the newly introduced condition (3). Theorem 2 (on the existence of PHL) is reformulated with emphasis on the most general nature of excitation. Theorem 3 (High-frequency behavior) is a generalized version of the theorem for extended memristors given in [3]. Theorems 4–7 are yet unpublished theorems that develop the findings presented in [23] and [24].

The example of a fluid meminerter analyzed and modeled in Section 3 was chosen for three practical reasons. The first reason was the topicality of the problem, as a large number of useful applications are emerging in the field of fluid mechanics and micromechanics [16]. The second reason was the opportunity to show how different processes taking place within a single device can be modeled using ideal, generic, or extended (*α*,*β*) elements. The third reason was the opportunity to present a practical example of an element that requires modification in the existing definition of extended memristors, memcapacitors, and meminductors. In this case, it is an extended memristor for modeling parasitic damping (20).

## 5. Conclusions

In this work, the class of extended and generic higher-order elements is defined as a natural generalization of hitherto established extended and generic variants of the memristor, memcapacitor, and meminductor. The definition allows a unified approach to the description of memristive, memcapacitive, and meminductive processes that are used in contemporary MEMS applications and related fields. At the same time, it provides an apparatus suitable for describing processes that have been less studied so far, such as meminertance. The story of the mechanical inerter [25] shows that even nowadays there are important discoveries of new elements, which by their constitutive relations embody some fundamental physical laws. Such elements greatly advance the possibilities of circuit synthesis and are a considerable aid in predictive modeling.

## Figures and Tables

**Table 1:** Uniform description of extended (e) and generic (g) elements according to definition (2).

Element	*y*	*u*	*e*: *P*(*x*,*u*); *g*: *P*(*x*)
Extended and Generic Memristor 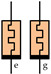	Voltage *v* = *v*^(0)^	Current *i* = *i*^(0)^	Resistivity *e: R*(*x*,*i*), *g*: *R*(*x*)
	Current *i* = *i*^(0)^	Voltage *v* = *v*^(0)^	Conductance *e: G*(*x*,*v*), *g: G*(*x*)
Extended and Generic Memcapacitor 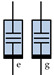	Charge *q* = *i*^(−1)^	Voltage *v* = *v*^(0))^	Capacitance *e: C*(*x*,*v*), *g: C*(*x*)
	Voltage *v* = *v*^(0))^	Charge *q* = *i*^(−1)^	Elastance *e: D*(*x*,*q*), *g: D*(*x*)
Extended and Generic Meminductor 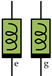	Flux *φ* = *v*^(−1)^	Current *i* = *i*^(0)^	Inductance *e: L*(*x*,*i*), *g: L*(*x*)
	Current *i* = *i*^(0)^	Flux *φ* = *v*^(−1)^	Reluctance *e:* *Λ*(*x*,*φ*), *g:* *Λ*(*x*)
Extended and Generic (*α*,*β*) element 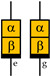	*y* = *v*^(*α*+1)^	*u* = *i*^(*β*+1)^	Common parameter *P* *e: P*(*x*,*i*^(*β*+1)^), *g*: *P*(*x*)
	*y* = *i*^(*β*+1)^	*u* = *v*^(*α*+1)^	Dual parameter *P_d_* *e: P_d_*(*x*,*v*^(*α*+1)^), *g*: *P_d_*(*x*)

**Table 2:** Electromechanical *F-i* and *F-v* analogies.

Mechanical Variables	Electrical Variables	
	*F-i*	*F-v*
Force	Current	Voltage
Momentum	Charge	Flux
TIM ^1^	TIC ^2^	TIF ^3^
Velocity	Voltage	Current
Displacement	Flux	Charge
Absement	TIF ^3^	TIC ^2^

^1^ Time Integral of Momentum. ^2^ Time Integral of Charge. ^3^ Time Integral of Flux

## Data Availability

Not applicable.

## Appendix A

* SPICE Model of Fluid EDamper

* For Transient Analysis only

**************************

* d1**—**Piston diameter

* d2**—**Piston rod diameter

* rh**—**Helical channel radius

* Ph**—**Helix pitch

* w**—**Piston width

* X0**—**Initial position of the piston

* rho**—**Fluid density

*

.SUBCKT EDamper Plus Minus PARAMS:

+ d1=50mm d2=20mm rh=6mm X0=-30mm Ph=14mm w=100mm rho=760

******************************************

* FORCE**—**VELOCITY PORT MODELING *

******************************************

* Ed voltage represents viscous friction force

* Ed current represents velocity

Ed Plus Minus value={I(Ed)*Rd(v(x),I(Ed))}

******************************************

* POSITION OF PISTON CALCULATION *

******************************************

* Ex voltage represents piston position

Ex X 0 value={SDT(I(Ed))+X0}

******************************************

* AUXILIARY *

******************************************

.PARAM Dhydro={2*rh*pi/(pi+2)} Rhydro={d1/2}

.PARAM c1= {0.004283*pi*rho*(d1^2-d2^2)^3*

+ sqrt(Ph^2+(pi*d1)^2)/Ph/rh^4/sqrt(Dhydro*Rhydro)}

.PARAM c2= {3*pi*rho*(d1^2-d2^2)^3/64/rh^4}

.FUNC Rd(position,velocity)={(c1*(w/2-position)+c2)*abs(velocity)}

*

.ENDS EDamper
